# Geographic Access to Primary Care and Racial and Ethnic Disparities in the Prevalence of Maternal Chronic Conditions 2020–2024

**DOI:** 10.5888/pcd23.260235

**Published:** 2026-10-15

**Authors:** Sheree Boulet, Lauren Costley, Kaitlyn Stanhope

**Affiliations:** 1 Department of Obstetrics and Gynecology, Boston University Chobanian & Avedisian School of Medicine, Boston, Massachusetts; 2 Wellstar Kennestone Regional Medical Center, Marietta, Georgia; 3 Department of Epidemiology, Rollins School of Public Health, Emory University, Atlanta, Georgia

## Abstract

**Introduction:**

Unequal access to preventive health care before and between pregnancies contributes to risk for adverse perinatal outcomes. Few studies have examined access to primary care as a contributor to racial and ethnic disparities in chronic disease prevalence among women having a live birth.

**Methods:**

We linked 2020–2024 natality data with Health Resources and Services Administration’s Area Health Resource Files and used spatial Bayesian conditional autoregressive models to estimate county-level associations between geographic access to primary care and prevalence of chronic conditions (obesity, hypertension, and diabetes) in 1,374 counties in the contiguous US. We defined geographic access to primary care using the Health Resources and Services Administration’s primary care Health Professional Shortage Area (HPSA) designation and the number of primary care providers per 100,000 persons in quartiles. We assessed racial disparities in outcomes by modeling the difference in rate differences and 95% CIs among non-Hispanic Black and non-Hispanic White women having a live birth, controlling for county characteristics.

**Results:**

Residing in a county designated as a primary care HSPA or with lower rates of density of primary care providers was associated with modestly increased prevalence of obesity and hypertension but not diabetes. However, across the chronic conditions, the disparity between non-Hispanic Black and non-Hispanic White women was generally consistent regardless of geographic access to primary care. For example, the rate difference between the 2 groups for obesity was 11.3 (95% CI, 10.5–12.0) per 100 births for primary care HPSAs and 9.9 (95% CI, 9.5–10.4) per 100 births for non–primary care HPSAs, while the adjusted β for the difference in rate differences was 0.005 (95% CI, −0.005 to 0.014).

**Conclusion:**

Geographic access to primary care did not attenuate racial disparities in the prevalence of chronic conditions among women who had a live birth.

**Summary**

**What is already known on this topic?**

Early detection and management before pregnancy of chronic conditions such as obesity, hypertension, and diabetes can reduce risks for adverse perinatal outcomes.

**What is added by this report?**

Among women having a live birth in the previous year, living in a county with less access to primary care in the year before delivery was associated with increased prevalence of obesity and hypertension. However, disparities in the prevalence of chronic conditions among non-Hispanic Black and non-Hispanic White women were largely constant, regardless of access to primary care.

**What are the implications for public health practice?**

Increasing geographic access to care may not be sufficient for reducing disparities in chronic disease burden among reproductive-aged women experiencing structural barriers to accessing primary and preventive care.

## Introduction

Pregnancy-related morbidity and mortality in the US have increased over the past 3 decades, with enduring racial and ethnic disparities in risk (1,2). Chronic conditions, including obesity, hypertension, and diabetes, increase risk of pregnancy complications. The prevalence of obesity, hypertension, and diabetes has increased over time, with growing numbers of women affected by multiple conditions (3,4).

Access to preconception health care and interpregnancy care is critical for improving perinatal outcomes because it affords an opportunity to address health risks before pregnancy, a time when physical and social exposures can have lasting effects on maternal and infant health (5). Current guidelines recommend routine prepregnancy screening and counseling during every clinical encounter for reproductive-aged women, including on topics related to nutrition and chronic disease management (5). More than 90% of preventive care visits for reproductive-aged women occur in the primary care setting (6), and disparities in receipt of essential services such as blood pressure and diabetes screening, particularly for women who are Black, lack health insurance, or have low family incomes, have been documented (7).

Geographic access to primary care improves uptake of preventive health services and health status in the general population, even after accounting for health care, socioeconomic, and demographic factors (8–10). Living in geographic areas with a shortage of health care providers is associated with increased rates of morbidity and mortality (10,11). However, little is known about the role of access to primary care in improving the detection, treatment, and control of maternal chronic disease, despite associations between prepregnancy health status and maternal morbidity risk (12). We used US natality data to investigate population-level associations between geographic access to primary care and racial and ethnic disparities in the prevalence of maternal chronic disease. We hypothesized that disparities in the prevalence of chronic conditions would be reduced among women living in areas with greater access to primary care.

## Methods

### Study population

We used data from the 2020–2024 National Center for Health Statistics restricted-use natality files, which included birth certificate information for all live births in the US and data on county residence at birth. We included births to resident women aged 15 to 44 years living in the contiguous US (n = 18,026,492) and excluded records where county of residence was unknown (n = 37,946). Alaska and Hawaii were excluded from analysis because geographic barriers to care differ in these states from barriers in the contiguous US. Alaska’s vast territories with limited road connectivity and communities accessible only by air or water present unique challenges. Hawaii consists of 137 islands, but most health care facilities are centrally located in Oahu, often requiring ferries or flights to access care. We aggregated the natality data by county Federal Information Processing Series (FIPS) code and year of delivery and merged the data file with the Health Resources and Services Administration’s (HRSA’s) Area Health Resources Files and data from the US Census Bureau’s Annual County Resident Population Estimates for 2020–2024 (13). We excluded data for 8 historical counties in Connecticut because they were replaced in 2022 with 9 planning regions and could not be linked across data sets using FIPS codes (14). HRSA’s Area Health Resources Files data included annual information on county-level primary care health provider shortage area (primary care HPSA) designation, number of primary care providers, percentage of persons below the federal poverty level, and Rural–Urban Continuum codes (15). We ascertained information on self-reported race and ethnicity from the birth certificate. To improve the stability of estimates, we aggregated county-level data across the 5-year study period and excluded counties with fewer than 50 births to non-Hispanic Black (Black) women or fewer than 50 births to non-Hispanic White (White) women. We used US Census data to estimate county-level population denominators for the density of primary care providers measure and to calculate the age and racial composition of women of reproductive age for each county (19). We calculated county-level age and racial composition by summing the total number of women in each age category and race and ethnicity category for each county during the study period and dividing it by the total number of women of reproductive age (15–44 y) living in each county.

### Measures

Our outcomes of interest were the average prevalence of prepregnancy obesity (body mass index ≥30 kg/m^2^), chronic hypertension, and diabetes as reported on the birth certificate. Body mass index was calculated using prepregnancy weight and height reported by the mother at time of delivery. Chronic hypertension and diabetes were reported by hospital staff using a checkbox to indicate the risk factor was present. We calculated the prevalence by summing the total number of women with the outcome for each county during the 5-year study period and dividing it by the total number of live births in the county for the same period.

Our exposure was geographic access to primary care, which we defined in 2 ways. First, we used the HRSA designation indicating that the entire county was a geographic primary care HPSA during any year of the study period. We did not consider partial primary care HPSAs for this study because they represent counties where only some areas have been designated as HPSAs. We did not have access to the more granular data needed to analyze those smaller jurisdictions. HRSA defines primary care providers as nonfederal physicians who provide direct patient care in general or family practice, general internal medicine, pediatrics, and obstetrics and gynecology. To be designated as a geographic primary care HPSA, counties must 1) be a rational area for the delivery of primary medical care (contiguous area consisting of established communities that have a travel time of more than 30 minutes to primary care services, accounting for terrain and public transit), 2) have a population to primary care provider ratio of 3,500 or more to 1 or 3,000 to 1 if unusually high primary care needs or insufficient provider capacity is present, and 3) be located near contiguous areas with excessively distant, inaccessible, or overutilized primary care providers (15,16). Second, we used data on counts of primary care providers to calculate the rate of primary care providers per 100,000 persons, categorized into quartiles. All exposures were lagged by 1 year before delivery year such that the exposures (residing in a primary care HPSA and density of primary care providers) were assessed in the year before the delivery took place.

### Statistical analysis

The unit of analysis for this ecological study was county of residence. We calculated the mean distribution of county-level birth and population characteristics across the study period (age at delivery, educational attainment, health insurance type, parity, percentage unemployment, percentage of families below the federal poverty level, racial and ethnic distribution of women aged 15–44 years, and the percentage of counties designated as rural). We determined rurality by using Rural–Urban Continuum Codes of 4 through 9 and dichotomized counties as rural or nonrural. Because our goal was to compare racial and ethnic differences in the prevalence of chronic conditions, we stratified the data by Black and White women who had a live birth and used empirical Bayes estimators to generate smoothed county-level estimates of the prevalence of each outcome of interest. We assessed spatial autocorrelation using Moran *I* (range, 0.28–0.33, *P* < .001 for all outcomes). For model building, we considered Queen, k-nearest neighbors, and sphere-of-influence contiguities and compared models using the sum of the log of the conditional predictive ordinate values, Watanabe–Akaike information criterion, and deviance information criterion. The Queen adjacency test performed best overall and was used for analysis, with an average of 4.2 neighbors. We categorized empirical Bayes smoothed estimates for each outcome into tertiles and created maps of the county level distributions, stratified by race.

We used Bayesian conditional autoregressive (CAR) negative binomial models with Besag, York, and Mollié priors and Queen adjacency to estimate adjusted rate ratios (ARRs) and 95% CIs for the association between primary care HPSA designation and quartiles of primary care provider density and each outcome of interest, adjusting for county-level measures of percentage of families below the federal poverty level, unemployment rate, rurality, the age and racial composition of women of reproductive age, and year of delivery. Bayesian CAR models account for spatial autocorrelation and provide shrinkage and smoothing of the effect estimates by borrowing information from nearby neighboring areas (17). We used negative binomial distribution to account for overdispersion. We evaluated multicollinearity using variance inflation factors, and all were less than 3.4.

To assess disparities between Black and White women, we used race-specific rates to calculate Black–White rate ratios and rate differences and estimated Bayesian linear CAR models with Besag, York, and Mollié priors and Queen adjacency for the association between geographic access to primary care (primary care HPSA and quartiles of provider density) and the difference between the Black–White rate differences for each outcome, adjusting for percentage of families below the federal poverty level, unemployment rate, rurality, age and racial composition of women of reproductive age, and year of birth. We calculated the difference in the Black–White rate differences by subtracting the Black–White rate difference for each outcome of interest for counties designated as primary care HPSAs from the Black–White rate difference for counties that were not designated as primary care HPSAs. Likewise, we calculated the difference in the Black–White rate differences by subtracting the Black–White rate difference for each outcome of interest for counties in quartile 1 through 3 from the Black–White rate difference for counties in the fourth quartile. The measure can be conceptualized as the difference in disparities associated with access to primary care providers.

We used Q–Q plots and Kolmogorov–Smirnov tests to confirm the normality of the residuals for the linear CAR models. We used SAS version 9.4 (SAS Institute Inc) and R version 4.5.0 (R Foundation) for analysis, including the R-INLA package for estimating Bayesian CAR models (18). This study was approved by Emory University’s Institutional Review Board (STUDY00003587) and was considered not human subjects research by the Boston Medical Center and Boston University Medical Campus Institutional Review Board. 

### Sensitivity analysis

To evaluate the impact of excluding counties with 50 or fewer births to Black or White women, we derived county-level maps depicting the distribution of primary care HPSAs, primary care provider density (quartiles), and proportion of White and Black women having a live birth (tertiles) among the full study population.

We also conducted aspatial analyses of individual-level data to assess the robustness of the spatial results. Using robust Poisson regression, we estimated the association between primary care HPSA and density of primary care providers and all outcomes of interest, controlling for age at delivery, year of delivery, rurality of county of residence, county percentage of families below the federal poverty level, and county unemployment rate. All covariates were measured at the individual level. The models included generalized estimating equations to account for clustering by county; thus, the effect estimates represent population-level effects for comparison with the spatial data.

## Results

Of the 3,099 counties in the dataset (after excluding 9 Connecticut counties), 1,374 had more than 50 live births to Black or White women during the study period and were included in the analysis. Counties designated as a primary care HPSA had a higher mean proportion of births to women aged 15 to 24 years than non–primary care HPSAs (34.3% [SD, 7.1%] vs 25.9% [SD, 9.1%]) (Table 1). The mean proportion of births to women with less than a high school education and Medicaid–enrolled deliveries was also higher in primary care HPSA counties than in non–primary care HPSA counties (13.0% [SD, 5.6%] vs 11.4% [SD, 5.2%] and 55.8% [SD, 14.4%] vs 43.5% [SD, 14.4%], respectively). In addition, primary care HPSA counties had a higher mean percentage of families below the federal poverty level than non–primary care HPSAs (14.2% [SD, 5.5%] vs 9.9% [SD, 4.4%]). A higher mean proportion of Black women of reproductive age were present in primary care HPSA counties than in non–primary care HPSA counties (32.9% [SD, 21.0%] vs 17.0% [SD, 15.4%]). More primary care HPSA counties than non–primary care HPSA counties were classified as rural (214 vs 36). Similarly, counties in the lowest quartile of primary care provider density had higher proportions of births to women aged 15 to 24 years, higher proportions of women with less than a high school education, more Medicaid-enrolled deliveries, higher percent unemployment, and more births to Black women compared with counties in the higher quartiles of primary care provider density. 

**Table 1:** County-Level Characteristics by Geographic Access to Primary Care, 1,374 Counties in the Contiguous US With >50 Births, National Center for Health Statistics Restricted-Use Natality Files 2020–2024^a^

Characteristic	Primary care health provider shortage area^b^		Primary care provider density (per 100,000)			
	Yes (n = 324)	No (n = 1,050)	1st quartile (lowest) (n = 344)	2nd quartile (n = 344)	3rd quartile (n = 343)	4th quartile (highest) (n = 343)
**Age, y**						
15–24	34.3 (7.1)	25.9 (9.1)	33.1 (7.6)	30.3 (8.2)	27.0 (8.4)	20.9 (8.4)
25–29	31.5 (2.4)	29.8 (3.7)	31.6 (2.4)	31.4 (2.2)	30.3 (2.9)	27.6 (4.6)
30–34	22.5 (4.1)	28.0 (5.9)	23.3 (4.4)	25.0 (5.1)	27.1 (5.5)	31.5 (5.6)
35–44	11.6 (3.0)	16.3 (6.1)	12.0 (3.3)	13.3 (3.9)	15.5 (5.2)	20.1 (7.0)
**Education**						
Less than high school	13.0 (5.6)	11.4 (5.2)	12.7 (5.3)	12.5 (5.7)	11.8 (5.7)	10.0 (4.2)
High school	38.1 (7.3)	29.3 (8.5)	36.8 (7.3)	33.6 (7.9)	30.3 (7.8)	24.7 (8.3)
Some college	30.9 (5.2)	28.2 (5.7)	31.2 (5.3)	30.0 (4.8)	28.9 (5.2)	25.0 (5.7)
Bachelor’s degree or more	17.6 (7.8)	30.3 (13.7)	18.8 (8.6)	23.2 (10.8)	28.2 (11.9)	39.0 (13.7)
**Health insurance**						
Private	36.2 (13.2)	48.1 (14.8)	38.3 (13.4)	43.2 (14.6)	45.7 (14.3)	54.0 (14.3)
Medicaid	55.8 (14.4)	43.5 (14.4)	53.3 (14.1)	48.6 (15.1)	45.1 (14.2)	38.4 (13.9)
Self	3.9 (4.0)	4.1 (3.5)	4.1 (3.9)	4.3 (4.2)	4.1 (3.5)	3.5 (2.8)
Other	3.6 (7.4)	3.7 (6.6)	3.6 (6.3)	3.4 (6.8)	4.2 (7.2)	3.4 (6.7)
Parity						
0	36.0 (2.6)	37.5 (3.2)	36.2 (2.6)	36.2 (2.6)	37.2 (3.0)	38.8 (3.4)
1	30.6 (2.2)	31.6 (2.2)	30.8 (2.2)	31.1 (2.0)	31.3 (2.2)	32.2 (2.2)
≥2	33.2 (3.6)	30.7 (4.4)	32.9 (3.6)	32.4 (3.8)	31.2 (4.3)	28.6 (4.5)
**Unemployment**	4.9 (1.3)	4.6 (1.2)	4.7 (1.1)	4.8 (1.4)	4.7 (1.2)	4.4 (1.0)
**Families below the federal poverty level**	14.2 (5.5)	9.9 (4.4)	13.0 (5.0)	11.5 (5.1)	10.3 (4.9)	8.6 (3.9)
**Race and ethnicity of women of reproductive age**						
American Indian/Alaska Native	1.4 (2.9)	1.4 (2.7)	1.5 (2.9)	1.5 (3.8)	1.3 (2.0)	1.2 (1.8)
Asian	1.4 (1.3)	4.3 (4.8)	1.5 (1.7)	2.3 (2.1)	3.9 (3.8)	6.8 (6.3)
Hispanic	8.5 (11.2)	13.6 (13.3)	10.0 (11.1)	11.7 (14.3)	15.3 (14.9)	12.6 (10.7)
Native Hawaiian/Pacific Islander	0.2 (0.3)	0.3 (0.3)	0.2 (0.2)	0.2 (0.4)	0.3 (0.4)	0.3 (0.4)
Non-Hispanic Black	32.9 (21.0)	17.0 (15.4)	27.3 (19.0)	20.8 (18.6)	17.7 (17.5)	17.2 (15.7)
Non-Hispanic White	57.4 (18.9)	66.1 (18.6)	61.6 (17.9)	65.7 (19.8)	64.0 (20.1)	64.9 (18.2)
**Rural,^c^** no. (%)	214 (66.0)	336 (32.0)	198 (57.6)	185 (53.8)	127 (37.0)	40 (11.7)

a All values are mean (SD) percentage unless otherwise indicated. Percentages may not add to 100 as the values represent the average distribution across all counties during the study period.

b County was ever designated as a primary care health provider shortage area, 2020–2024.

c County was ever designated as rural, 2020–2024.

Overall, the prevalence of all chronic conditions was higher among Black women who had a live birth than among White women who had a live birth (Figure 1). The prevalence of all chronic conditions was higher in the southern and southwestern regions, regardless of race (Figure 1). Primary care HPSAs were more concentrated in the southern and midwestern regions, with lower provider density showing similar patterns (Figure 2). The Southeast had a higher mean percentage of births to Black women, whereas the Northeast and Northwest had a higher mean percentage of births to White women (Figure 2).

**Figure 1:**
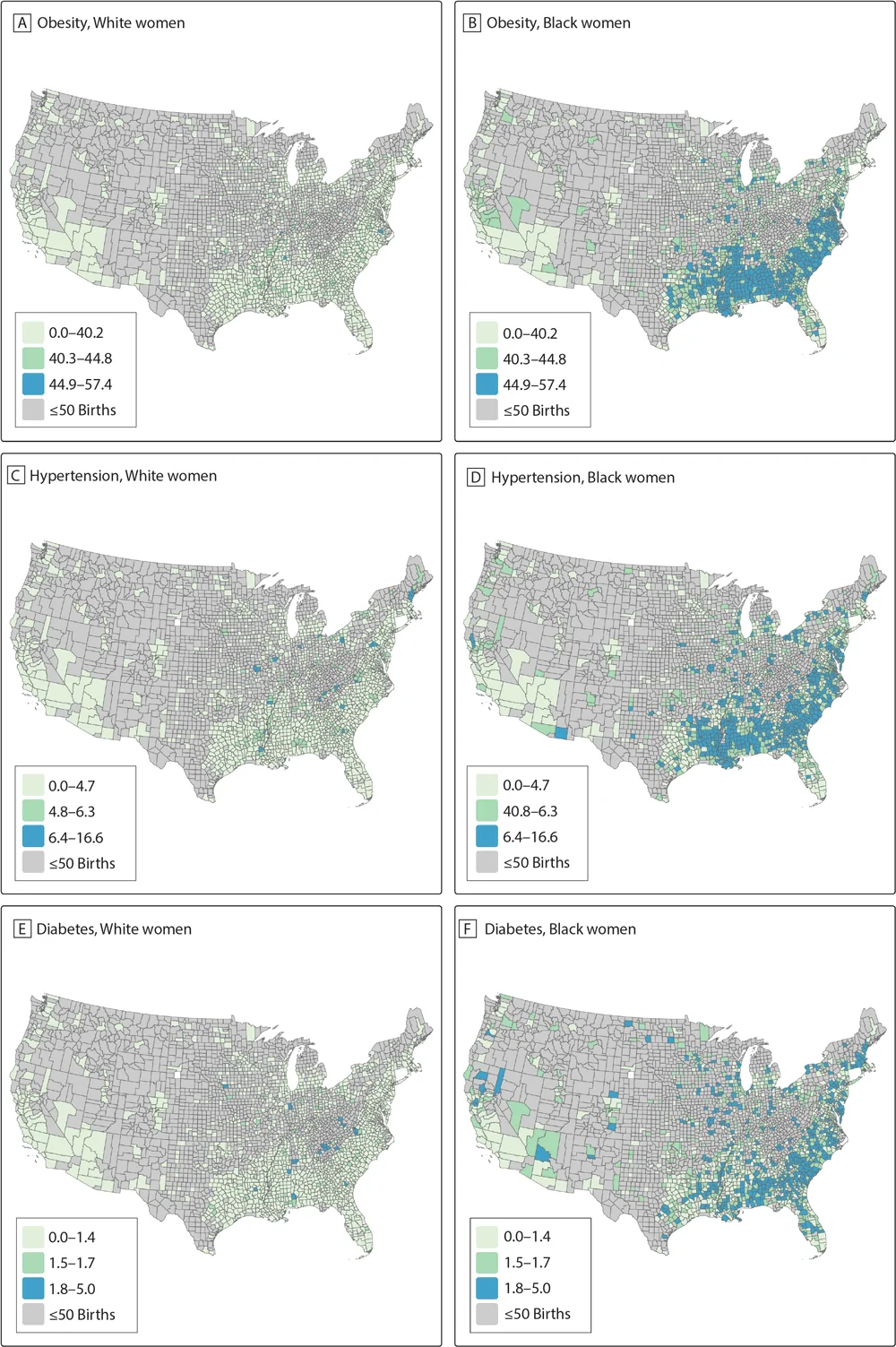
Empirical Bayes smoothed distribution of mean county-level prevalence of prepregnancy obesity, chronic hypertension, and diabetes among non-Hispanic White and non-Hispanic Black women having a live birth, National Center for Health Statistics Restricted-Use Natality Files, US, 2020–2024. Maps show 1,374 counties in the contiguous US with >50 births. A) Prepregnancy obesity among non-Hispanic White women having a live birth. B) Prepregnancy obesity among non-Hispanic Black women having a live birth. C) Chronic hypertension among non-Hispanic White women having a live birth. D) Chronic hypertension among non-Hispanic Black women having a live birth. E) Diabetes among non-Hispanic White women having a live birth. F) Diabetes among non-Hispanic Black women having a live birth.

**Figure 2:**
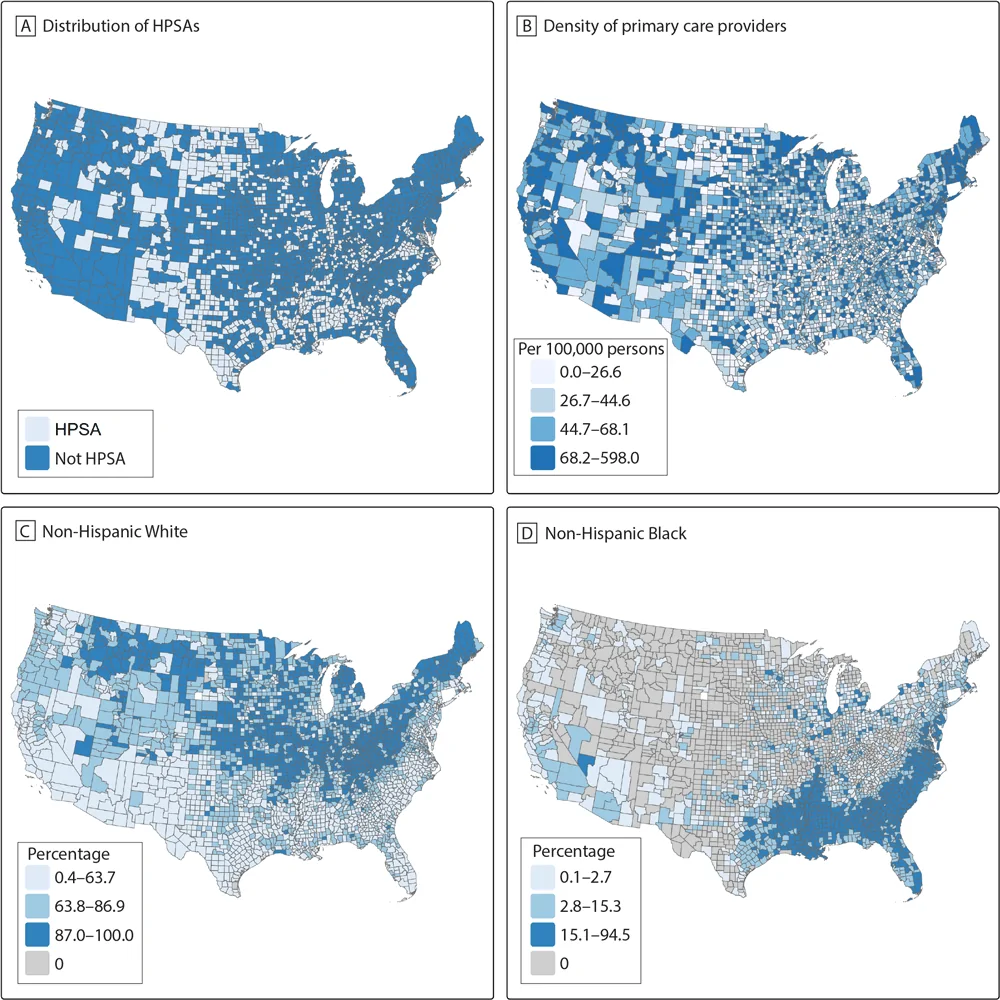
Distribution of primary care health provider shortage areas, density of primary care providers, and live births to non-Hispanic White and non-Hispanic Black women, National Center for Health Statistics Restricted-Use Natality Files, US, 2020–2024. A) Primary care HPSAs. B) Density of primary care providers per 100,000 persons (quartiles). C) Percentage of live births to non-Hispanic White women (tertiles). D) Percentage of live births to non-Hispanic Black women (tertiles). Abbreviation: HPSA, health provider shortage area.

In the adjusted models, primary care HPSAs were significantly associated with increased prevalence of obesity (adjusted rate ratio [ARR], 1.04; 95% CI, 1.02–1.06), as were areas of lower primary care provider density (quartile [Q]1 vs Q4 ARR, 1.16; 95% CI, 1.14–1.18, Q2 vs Q4 ARR, 1.14; 95% CI, 1.12–1.16; Q3 vs Q4 ARR, 1.09, 95% CI, 1.07–1.11) (Table 2). Primary care HPSAs were not significantly associated with hypertension and diabetes prevalence after adjustment (ARR, 1.04; 95% CI, 0.99–1.10 and ARR, 1.01; 95% CI, 0.96–1.07, respectively). After adjustment, the lowest quartile of primary care provider density was modestly associated with hypertension (Q1 vs Q4 ARR, 1.07; 95% CI, 1.01–1.14).

**Table 2:** Association Between Geographic Access to Primary Care and Maternal Chronic Conditions,1,374 Counties in the Contiguous US,^a^ National Center for Health Statistics Restricted-Use Natality Files 2020–2024

Exposure	Prepregnancy obesity			Chronic hypertension			Diabetes		
	Mean prevalence per 100 births	RR (95% CI)	ARR^b^ (95% CI)	Mean prevalence per 100 births	RR (95% CI)	ARR^b^ (95% CI)	Mean prevalence per 100 births	RR (95% CI)	ARR^b^ (95% CI)
**Primary care health provider shortage area**									
Yes	40.4	1.13 (1.10–1.15)	1.04 (1.02–1.06)	4.8	1.22 (1.15–1.28)	1.04 (0.99–1.10)	1.3	1.09 (1.04–1.14)	1.01 (0.96–1.07)
No	34.1	[Reference]	[Reference]	3.5	[Reference]	[Reference]	1.5	[Reference]	[Reference]
**Primary care provider density per 100,000 persons (quartile)**									
Quartile 1 (lowest)	39.6	1.23 (1.20–1.26)	1.16 (1.14–1.18)	4.4	1.20 (1.13–1.27)	1.07 (1.01–1.14)	1.40	1.06 (1.01–1.12)	1.00 (0.95–1.06)
Quartile 2	37.4	1.19 (1.16–1.21)	1.14 (1.12–1.16)	4.0	1.10 (1.04–1.17)	1.05 (1.00–1.11)	1.30	1.04 (0.99–1.10)	1.01 (0.95–1.06)
Quartile 3	34.6	1.12 (1.09–1.14)	1.09 (1.07–1.11)	3.5	1.02 (0.97–1.09)	1.00 (0.95–1.05)	1.3	1.03 (0.98–1.08)	1.01 (0.96–1.06)
Quartile 4 (highest)	30.8	[Reference]	[Reference]	3.5	[Reference]	[Reference]	1.3	[Reference]	[Reference]

Abbreviation: ARR, adjusted rate ratio.

a Counties with ≥50 births to non-Hispanic Black and non-Hispanic White women.

b Estimated by using spatial conditional autoregressive models with Besag, York, and Mollié priors and adjusted for percentage of families below the federal poverty level, unemployment rate, rurality, age and racial composition of women of reproductive age, and year of delivery.

The results of the aspatial analysis of individual-level data were largely consistent with the results of the spatial analysis. Among 17,585,981 births with nonmissing data on obesity, hypertension, or diabetes, living in a primary care HPSA was associated with increased prevalence of obesity (ARR, 1.06; 95% CI, 1.04–1.09). The 3 lower quartiles of primary care provider density were also associated with increased risk for obesity when compared with the highest quartile (Q1 vs Q4 ARR, 1.19; 95% CI, 1.16–1.22, Q2 vs Q4 ARR, 1.15; 95% CI, 1.11–1.19; Q3 vs Q4 ARR, 1.10, 95% CI, 1.06–1.14). We did not find significant associations between either measure of access to primary care and hypertension or diabetes after adjustment.

Overall, the prevalence of all 3 chronic conditions was higher among Black women than among White women across all strata of primary care provider access. For example, the Black–White rate difference for obesity was 11.3 (95% CI, 10.5–12.0) per 100 births for primary care HPSAs and 9.9 (95% CI, 9.5–10.4) per 100 births for non–primary care HPSAs (Table 3). In the adjusted model assessing racial differences in rates of obesity, the β for the difference in Black–White rate differences was 0.005 (95% CI, −0.005 to 0.014), indicating that the average Black–White rate difference was only slightly larger in primary care HPSAs compared with non–primary care HPSAs. Similarly, Black race was associated with 3 excess cases of hypertension per 100 births (rate difference, 3.2; 95% CI, 2.9–3.5) for primary care HPSA counties and 2 excess cases of hypertension per 100 births (rate difference 2.3; 95% CI, 2.1–2.4) in non–primary care HPSA counties. However, the adjusted beta for the difference in Black–White rate differences was also small (0.004; 95% CI, 0.001–0.007). We found some evidence of reduced racial disparities for the lower quartiles of primary care provider density relative to the highest quartile for all outcomes of interest, although the βs were modest.

**Table 3:** Association Between Geographic Access to Primary Care and Racial Disparities in Maternal Health Indicators, 1,374 Counties in the Contiguous US^a^, National Center for Health Statistics Restricted-Use Natality Files 2020–2024

Exposure	Prepregnancy obesity			Chronic hypertension			Diabetes		
	Black: White RR (95% CI)	Difference in Black: White rate^b^ (95% CI)	Adjusted^b^ β (95% CI) for the difference in rate differences	Black: White RR (95% CI)	Difference in Black: White rate^b^ (95% CI)	Adjusted^b^ β (95% CI) for the difference in rate differences	Black: White RR (95% CI)	Difference in Black: White rate^b^ (95% CI)	Adjusted^c^ β (95% CI) for the difference in rate differences
**Primary care health provider shortage area**									
Yes	1.32 (1.30 to 1.34)	11.30 (10.50 to 12.00)	0.005 (−0.005 to 0.014)	1.97 (1.86 to 2.08)	3.17 (2.87 to 3.46)	0.004 (0.001 to 0.007)	1.92 (1.71 to 2.14)	0.69 (0.55–0.83)	−0.0003 (−0.001 to 0.002)
No	1.37 (1.35–1.39)	9.92 (9.49–10.40)	[Reference]	1.80 (1.75 to 1.85)	2.28 (2.15 to 2.41)	[Reference]	1.89 (1.81 to 1.97)	0.73 (0.67 to 0.79)	[Reference]
**Primary care provider density per 100,000 persons (quartile)**									
Quartile 1 (lowest)	1.30 (1.27 to 1.32)	10.30 (9.59 to 11.10)	−0.020 (−0.031 to −0.009)	1.98 (1.87 to 2.09)	3.06 (2.78 to 3.33)	0.002 (−0.002 to 0.006)	1.88 (1.70 to 2.06)	0.69 (0.55 to 0.83)	−0.0003 (−0.002 to 0.001)
Quartile 2	1.28 (1.25 to 1.30)	9.20 (8.50 to 9.89)	−0.024 (−0.034 to −0.014)	1.74 (1.67 to 1.82)	2.34 (2.10 to 2.58)	−0.002 (−0.006 to 0.001)	1.88 (1.70 to 2.07)	0.70 (0.59 to 0.81)	−0.0002 (−0.002 to 0.001)
Quartile 3	1.31 (1.28 to 1.35)	9.19 (8.43 to 9.95)	−0.024 (−0.034 to −0.014)	1.74 (1.65 to 1.83)	2.12 (1.89 to 2.35)	−0.004 (−0.007 to 0.0002)	1.90 (1.74–2.05)	0.68 (0.58 to 0.79)	−0.0007 (−0.002 to 0.001)
Quartile 4 (highest)	1.54 (1.49 to 1.60)	12.20 (11.50 to 13.00)	[Reference]	1.90 (1.81 to 1.99)	2.43 (2.21 to 2.65)	[Reference]	1.94 (1.83–2.05)	0.81 (0.71 to 0.90)	[Reference]

Abbreviation: RR, rate ratio.

a Counties with ≥50 births to non-Hispanic Black and non-Hispanic White women.

b Per 100 births.

c Estimated by using spatial conditional autoregressive models with Besag, York, and Mollié priors and adjusted for percentage of families below the federal poverty level, unemployment rate, rurality, age and racial composition of women of reproductive age, and year of delivery.

## Discussion

In this study of county-level associations between geographic access to primary care and racial and ethnic disparities in maternal chronic conditions, we found that living in a county designated as a primary care HPSA or with lower density of primary care providers was associated with modestly increased rates of obesity and hypertension but not diabetes. The prevalence of obesity, hypertension, and diabetes was consistently higher among non-Hispanic Black versus non-Hispanic White women, and geographic access to primary care providers did not appear to appreciably attenuate these disparities, suggesting that other factors, including social, structural, and environmental determinants of health, contribute to the persistent differences in chronic disease prevalence.

We found that rates of chronic conditions were highest in the southeastern region of the US, which is consistent with patterns observed in other analyses of the general population (20). The high burden of disease in this region correlates with high proportions of Black women of reproductive age and is likely due in part to health inequities caused by structural racism and discrimination, mutually reinforcing systems of oppression that influence health through policies, practices, and behaviors that systematically obstruct access to health-promoting resources for racial groups considered inferior (21). Our results also indicate that counties designated as primary care HPSA or having low primary care provider density had greater proportions of deliveries to mothers who were enrolled in Medicaid and who identified as non-Hispanic Black than non–primary care HPSA counties. Those counties also had higher rates of poverty and were more often classified as rural. Comparable patterns of poverty, rurality, and other measures of social vulnerability in primary care HPSA counties have been reported and have been similarly attributed to underlying structural factors (22).

A robust body of literature suggests that access to primary care contributes to improved population health, lower mortality rates, and reduced health disparities (8–11). We found that poorer geographic access to primary care was associated with a higher prevalence of obesity and hypertension; however, effect estimates were small, and we found no association with diabetes. In addition, racial disparities in chronic conditions in our study were largely constant, regardless of access to primary care. The relationship between access to a provider and use of health care is complex, with geographic proximity representing only a single factor. Findings from a recent study demonstrated weak correlations between primary care physician supply and use of health care among older adults, challenging the prevailing assumption that increasing the number of physicians in an area will inevitably lead to improved uptake of care (23). A host of structural, institutional, and individual-level determinants such as health insurance coverage, difficulty in scheduling appointments, long wait times, lack of racially concordant and language-concordant providers, stigma, implicit bias among health professionals, absence of social support, and poor provider–patient relationships have also been shown to influence women’s uptake of primary care services but were not assessed in this analysis (24,25). Our results are consistent with another study, which found that county-level preterm birth severity (defined as lower gestational age at birth) among Black mothers was associated with structural barriers related to health insurance status, supply of Black physicians, and availability of publicly funded contraceptives but not residency in a primary care health provider shortage area (26). Black women in the study were also overrepresented in primary care HPSAs and areas with low access to primary care providers.

### Limitations

Our study is subject to several limitations. This ecological analysis used aggregate data and cannot be used to infer associations at the individual level. However, findings from the individual-level aspatial analysis supported the aggregate results. We used primary care HPSA and density of primary care providers as proxy indicators of geographic access to primary care. Measures such as vehicle access, driving distance, travel time, or transportation infrastructure may better reflect geographic barriers to accessing care (27). Our findings are not generalizable to Alaska, Hawaii, and 9 planning regions in Connecticut because they were excluded from the analysis. Because primary care HSPA designation is correlated with population characteristics such as rurality, controlling for rural designation may result in overadjustment bias. However, we found no evidence of multicollinearity among the covariates. Reverse causality is also possible if areas with a high prevalence of chronic conditions were more likely to be designated as primary care HPSAs due to insufficient capacity. We used county as the unit of analysis when smaller areas such as census tracts or blocks may be more accurate measures of geographic proximity. We did not examine many other factors that influence access to health care, such as affordability, accommodation (including hours of operation, appointment availability, and access to language services), and patient preferences. We aggregated data over a 5-year period and excluded 1,725 counties due to sparse births, which could mask temporal trends and affect generalizability. In addition, some counties did not share a common border with other counties, which could result in less reliable estimates (28). We did not explore other types of primary care providers (eg, nurse practitioners, physician assistants), who often provide a substantial proportion of care, particularly in rural areas. Physicians who were federal employees (eg, working in Veterans Health Administration facilities) were not included in the analysis because HPSA designation is intended to measure civilian access to health care. Finally, chronic conditions may be underreported on birth certificates, and validity could be systematically worse for areas with primary care provider shortages, thereby biasing the estimates toward the null (29).

### Conclusion

Primary care is considered the foundation of an effective health system because it can address most health problems present in a population (9). Women’s preventive services are a key element of primary care, and include reproductive life planning, contraceptive services, detection and management of chronic diseases, health promotion, immunizations, and mental health services, all of which improve women’s health status across the lifespan (30). Although access to health care improves health status and outcomes for women of reproductive age, the mere geographic availability of primary care physicians may not be sufficient to overcome long-standing disparities in chronic health conditions. Indeed, studies of the impacts of Medicaid expansion under the Affordable Care Act suggest that while insurance coverage and receipt of women’s preventive services increased overall, racial, ethnic, and socioeconomic gaps in use of reproductive services and health outcomes persisted (31,32). Despite increasing awareness of maternity care deserts as drivers of pregnancy-related morbidity (33), an understanding of the contribution of access to primary care, including primary care HPSA designation, is needed. Future studies can determine whether access to primary care improves control of chronic conditions before pregnancy and reduces risk for adverse pregnancy outcomes. Policy–focused solutions that aim to dismantle the structural drivers of maternal health disparities are necessary for promoting equitable access to health care and improving preconception health and pregnancy outcomes.
